# Sperm-duct gland content increases sperm velocity in the sand goby

**DOI:** 10.1242/bio.037994

**Published:** 2019-03-05

**Authors:** Leon Green, Charlotta Kvarnemo

**Affiliations:** 1 Department of Biological and Environmental Sciences, University of Gothenburg, Box 463, SE-405 30 Göteborg, Sweden; 2 The Linnaeus Centre for Marine Evolutionary Biology, University of Gothenburg, SE-405 30 Gothenburg, Sweden

**Keywords:** Accessory glands, Adaptations, Ejaculate components, Fertilization, Reproduction, Spermatozoa

## Abstract

Sperm performance is often tightly linked to male reproductive success. In many demersal gobiid fishes, the male attaches sperm embedded in a mucus produced by sperm-duct glands to the nest substrate before spawning takes place. Sperm are activated as the mucus and embedded gland content dissolve into the water. To test the importance of gland content on sperm function in *Pomatoschistus minutus*, a marine fish with external fertilization, we used a paired experimental design, with spermatozoa tested with and without sperm-duct gland content mixed into seawater. We measured sperm velocity, percentage of motile sperm and sperm viability over time. Sperm were found to swim 7.3% faster when gland content was mixed in the seawater. Percentage motile sperm was unaffected by the gland content. Sperm viability in seawater exceeded 24 h, but was unaffected by the gland content. An increase in sperm velocity of similar magnitude as found here has been shown by others to increase fertilization success. Since velocity-boosting properties of sperm-duct gland content have now been found in three distantly related goby species, this trait appears to be conserved across the Gobiidae family and may aid in reproduction across a range of species and environments.

This article has an associated First Person interview with the first author of the paper.

## Abstract (precis)

**Summary:** In sperm, velocity is often traded off for viability. However, we show here that in sand gobies, sperm-duct gland content increase velocity without reducing viability, thus mitigating such a trade-off.

## INTRODUCTION

Sperm are often viewed as short-lived DNA vectors, with an inverse relationship (i.e. a trade-off) between their velocity and viability (Ball and Parker, 1996; Møller, 1998; Levitan, 2000; but see Snook, 2005). This trade-off is expected to arise because both velocity and viability require energy (Levitan, 2000). When this is the case, any increase in one trait would come at the expense of the other, and it is then the optimal combination of the two traits that is under selection (Ball and Parker, 1996). However, both velocity and viability of sperm, as well as other traits, such as the percentage of sperm that are motile, can be affected by characteristics of the close environment. In particular, the non-gametic content of ejaculate is increasingly being recognized as an important factor influencing reproductive success in many taxa, for example in insects and birds (Cameron et al., 2007; Cornwallis and O'Connor, 2009; Simmons and Lovegrove, 2017). Content is typically produced by testes or glands nearby testes, and may modulate the chemical micro-environment of the eggs and sperm (Poiani, 2006; Perry et al., 2013). It has been shown that such non-gametic ejaculate components can activate, energize and protect sperm, supress fungal and microbial activity, and affect female oviposition rate and receptivity in a range of species from *Drosophila* to humans (Chapman et al., 1995; Chapman, 2001; Poiani, 2006; Giacomello et al., 2008; Fitzpatrick and Lüpold, 2014). Despite a growing awareness of the importance of ejaculate content other than sperm, we still know relatively little about the effect of such substances during reproduction, especially among animals with external fertilization.

Among fish, blennies (Giacomello et al., 2006), sculpins (Petersen et al., 2004) and gobies have accessory organs close to the testes called sperm-duct glands (SDGs) that contribute to the ejaculate content (Miller, 1984; also referred to as seminal vesicles, e.g. Fishelson, 1991). Among gobies, nest-holding males cover the nest walls with SDG-derived mucus trails that are embedded with sperm before females lay their eggs (Miller, 1984). Sperm are then released and become activated as the mucus dissolves and the sperm get in contact with the surrounding seawater mixed with SDG content (Marconato et al., 1996; Ota et al., 1996; Mazzoldi et al., 2000). This strategy occurs in addition to subsequent regular ejaculation over the eggs (Marconato et al., 1996) although it is unclear to what extent the SDG content contributes to the ejaculate.

Sperm competition is common among gobies (Magnhagen, 1999; Mazzoldi et al., 2000; Jones et al., 2001a,b; Poli et al., 2018). As females often take hours to deposit their eggs, this dual mode of sperm release likely reduces the male trade-off between fertilization and nest-guarding against sneaker males (Marconato et al., 1996; Scaggiante et al., 1999). In addition, a slow release of small ejaculates has been linked to increased fertilization success in an externally fertilizing polychaete and this may apply to fish as well (Olito and Marshall, 2018). Sperm of externally spawning fishes typically have a short functional life, that in most cases is limited to minutes or even seconds (Cosson et al., 2008). Goby sperm appear to be an exception (Scaggiante et al., 1999; Locatello et al., 2007), with sperm in some species still being motile after 3 days in seawater (sand goby, *Pomatoschistus minutus*, Pallas 1770, painted goby, *P. microps*, Krøer 1838, and two-spotted goby, *Gobiusculus flavescens*, Fabricius 1779, C. Kvarnemo et al., unpublished data).

The physiological properties of the SDG content was studied during the early days of comparative physiology (Young and Fox, 1937), but research on their ecological context came much later. SDG content in Gobiidae have been demonstrated to increase velocity and viability of sneaker males when tested in the presence of nest holding males SDG content (Locatello et al., 2013; Poli et al., 2018). Goby SDG content have also been shown to limit the growth of bacteria (Giacomello et al., 2008). In this experimental study, we used a paired design and compared sand goby sperm tested with and without SDG content in the water. We focused on breeding coloured males and examined sperm velocity and percent motile sperm (using computer assisted sperm analysis), and sperm viability, measured as percentage of live sperm <10 min and 24 h (using cell-staining methods). Through this study we aim to establish a baseline for the effect of SDG content on sperm velocity, percent of motile sperm and viability in the sand goby, as this is so far unknown. Considering the wide use of this species as a study organism for evolution and ecology (e.g. Jones et al., 2001a; Takegaki et al., 2012; Svensson et al., 2017), an improved understanding of its demersal spawning traits is of value.

For detailed procedures and definitions, please see the Materials and Methods and Supplementary information.

## RESULTS AND DISCUSSION

No difference was found in the percent of motile sperm [generalized linear mixed effects model, χ^2^ (1)=0.1374, *P*=0.7109], detection threshold [generalized linear mixed effects model, χ^2^ (1)=0.8671, *P*=0.3518], or number of tracked sperm [generalized linear mixed effects model, χ^2^ (1)=2.5473, *P*=0.1105], which supports the sampling methodology. To control for a potential effect of sperm numbers on velocity, a general linear model analysis of covariance was performed with VCL as dependent variable, treatment as factor and percent motile sperm as covariate. Non-significant interactions (*P*>0.05) were deleted from the model. Despite heterogeneous variances, the viability data were analysed with a repeated measures general linear model with both time and treatment as repeated measures for each individual replicate.

**Fig. 1.**
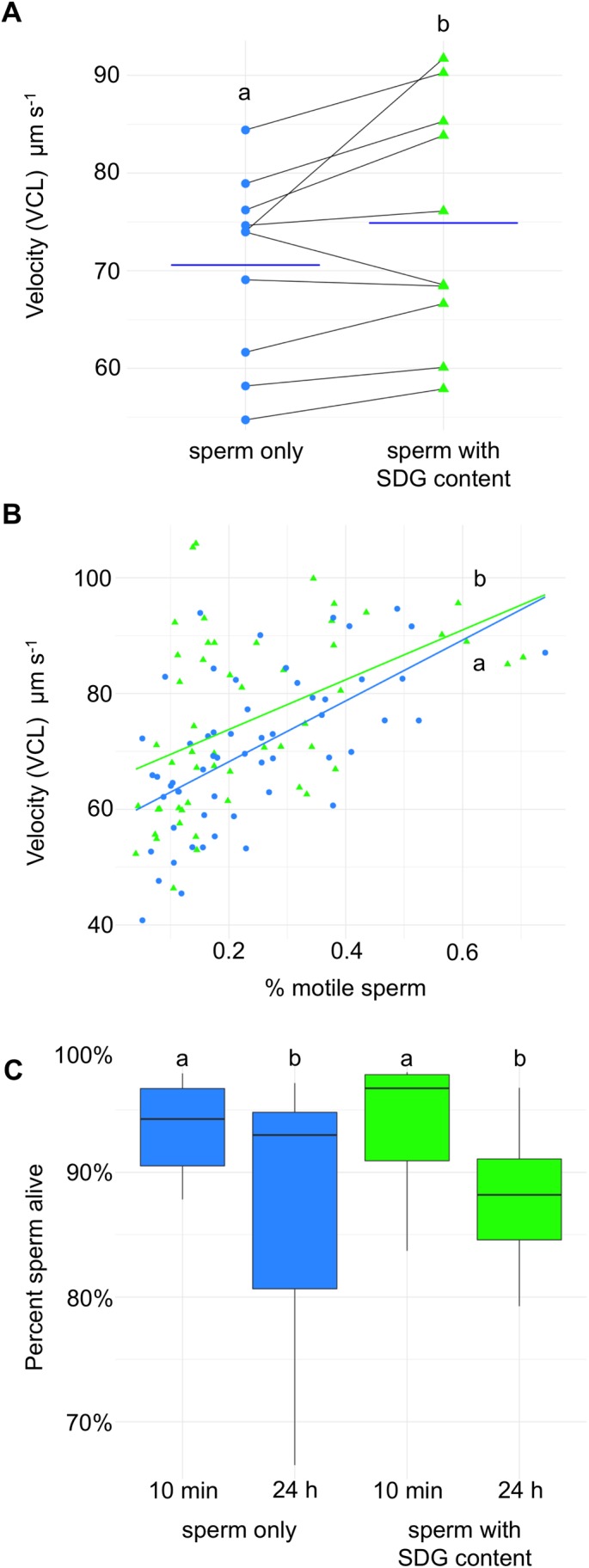
**Effects from SDG treatment on sperm traits.** Symbols show sperm from 10 males tested without (sperm only, blue dots) or with SDG content (sperm with SDG content, green triangles Δ) in seawater (see Materials and Methods for further details). Different letters show statistically significant differences (see Results and Discussion for all test values). Horizontal bars show means. (A) Sperm velocity increases in SDG content [generalized linear mixed effects model, χ^2^ (1)=5.475, *P*=0.019] shown as curvilinear velocity (VCL μm s^−1^). Each data point is the average of six technical replicates (*n*=10). (B) The percentage of motile sperm affects sperm velocity [general linear model, percentage of motile sperm (covariate): *F*_1,106_=40.866, *P*<0.001]. However, sperm velocity (VCL μm s^−1^) was still significantly higher for sperm with SDG content when controlling for percentage of motile sperm [general linear model, treatment (factor): *F*_1*,*106_=5.125, *P*=0.026], including all technical replicates (*n*=60). (C) The proportion of live sperm dropped slightly after 24 h compared to right after sampling (<10 min), but the drop was similar in the two treatments [general linear model, time (repeated): *F_1,9_*=8.92, *P*=0.015] (*n*=10).

We found that the treatment with content from the sperm-duct glands significantly increased the velocity of sperm in the sand goby [tested with SDG content mean±s.e.m. VCL: 75.38±2.07 μm s^−1^; tested without SDG content mean±s.e.m. VCL: 70.26±1.70 μm s^−1^; generalized linear mixed effects model, χ^2^ (1)=5.475, *P*=0.019, Fig. 1A]. Sperm tested with SDG content in the seawater showed an average increase in velocity by 5.12 μm s^−1^ (7.3%) compared to sperm tested without SDG content. Sperm velocity was still significantly different between treatments when controlling for the percentage of motile sperm [general linear model, treatment (factor): *F*_1*,*106_=5.125, *P*=0.026; percentage of motile sperm (covariate): *F*_1,106_=40.866, *P*<0.001] (Fig. 1B). The percentage of motile sperm was unaffected by treatment [generalized linear mixed effects model, χ*^2^*(1)=0.1374, *P*=0.7109].

Comparing the proportion of sperm that were alive after 10 min and 24 h, time had a significant effect on the viability of the sperm, but no statistically significant effect of treatment or interaction between time and treatment was found [general linear model, time (repeated): *F_1,9_*=8.92, *P*=0.015; treatment (repeated): *F_1,9_*=0.10, *P*=0.76; time*treatment: *F_1,9_*=0.03, *P*=0.87] (Fig. 1C). In both treatments, sand goby sperm showed a viability of over 86% of the sperm still alive after 24 h exposure to seawater.

Sperm velocity is directly linked to increased fertilization success in many taxa (Gage et al., 2004; Snook, 2005; Gasparini et al., 2010), and an increase in velocity of 5% can increase the relative fertilization success of a male by 5–6% (5% for an external fertilizer: from fig. 4C in Gallego et al., 2013; 6% for an internal fertilizer: from fig. 1 in Gasparini et al., 2010, value obtained through manually scaled measurements using GraphClick from Arizona Software). Such improved fertilization success is likely to have an important effect on male fitness, whether reproduction occurs under sperm competition or sperm limitation, both of which are common among externally fertilizing fish (including gobies) (Levitan, 1998; Petersen and Warner, 1998). Therefore, the increase in sperm velocity by 7.3% found in our study is expected to have a fitness effect through the males' fertilization success.

Viability is typically considered less important than velocity since fertilization during spawning in most species takes place within seconds of ejaculation (Cosson et al., 2008). Our results show that sand gobies are able to ‘boost’ sperm swimming speed through accessory substances without any measurable negative effect on viability. Levitan (1998, 2000) has suggested that long sperm viability would evolve as an adaptation to sperm limitation, whereas fast but short-lived sperm would evolve under sperm competition. Since sperm competition is well documented in sand gobies, the current result does not fit this picture. Instead, we suggest that the long lifespan of sand goby sperm may be an adaptation to ensure continued sperm function when egg deposition lasts one to several hours (Svensson and Kvarnemo, 2005). Considering that two to six females typically spawn sequentially in one nest (Jones et al., 2001a), the whole session can last even longer. Though the time window for fertilization of sand goby eggs has not been tested experimentally, other species' eggs remain fertilizable for several hours, enabling competition from parasitically spawning males, as found in grass goby (*Zosterisessor ophiocephalus*, Pallas 1814) and their close relative, the black goby (*Gobius niger*, Linnaeus 1758) (Mazzoldi, 1999; Scaggiante et al., 1999; Pilastro et al., 2002; Locatello et al., 2007).

The otherwise short lifespan of fish sperm in general has been attributed to an inability to handle osmotic change when ejaculated into an environment of different osmolality (Alavi and Cosson, 2006). However, sand goby sperm tolerate a wide range of osmolality and within the geographic range of the species, different populations are able to spawn successfully in salinities ranging from 35–3 practical salinity units (PSU) (Fonds and Van Buurt, 1974; Svensson et al., 2017). Hence, a general tolerance of an osmotically challenging environment may potentially explain the unusually long lifespan of the sperm.

Alternative reproductive tactics were not a focus of this study; yet, their role for SDG evolution makes them relevant to discuss in this context. As expected from theory (Parker, 1990; Parker et al., 1997), sneaker males of gobiid species typically have very large testes compared to nest-holding males, but they also have small SDGs (Kvarnemo et al., 2010; Locatello et al., 2013). In the sand goby, a distinct ‘sneaker morph’ is present. These males lack breeding colour and have testes three to four times the size of breeding coloured males, but smaller SDGs (approximately one fourth the size of breeding coloured males; Kvarnemo et al., 2010). Similar patterns are found in e.g. black goby (Rasotto and Mazzoldi, 2002) and grass goby (Scaggiante et al., 1999). In the black goby, sperm of sneaker males survive better over time, swim faster and have more adenosine triphosphate content than the sperm of nest-holding males, as tested without the aid of SDG content (Locatello et al., 2007; Poli et al., 2018). In the grass goby, while SDG function of nest-holders is mainly to produce mucus, in sneaker males it is primarily a sperm storage organ (Scaggiante et al., 1999). Nevertheless, both nest-holding and sneaker males are able to produce sperm trails (Mazzoldi et al., 2000). Sneaker males produce white sperm trails with many times higher sperm concentration than the opaque trails of nest-holding males (Mazzoldi et al., 2000). Furthermore, sperm velocity and fertilization success increase when the sneaker male sperm are exposed to seminal fluid from nest-holders (here: stripped fluid from testes and SDGs, with sperm removed), whereas for nest-holders the opposite is true (Locatello et al., 2013). In black gobies, however, seminal fluid increases sperm velocity of nest-holders, but not of sneaker males (Poli et al., 2018). In some populations of the sand goby, sneaker-morph males represent 10% of all males (Kvarnemo et al., 2010). However, parasitic spawnings also occur by other nest-holding males (Singer et al., 2006). Studies have shown close to 50% of broods to be partly fertilized by a male other than the nest-holder, independent of nest site availability (Jones et al., 2001b). Consequently, sperm competition is common in this species. Sneakers can change into nest-holders and develop breeding colouration (Takegaki et al., 2012). During this change, the SDG size increases, while testes size does not change, pointing to the importance of SDGs for the nest-holding reproductive tactic (Takegaki et al., 2012). Similar results from black goby indicate that plasticity in alternative reproductive tactics could be common among gobies with similar reproductive systems (Immler et al., 2004).

Our results are thus mirrored in grass and black goby, where SDG content also has a positive effect on sperm performance (Locatello et al., 2013; Poli et al., 2018). Since the genera *Zosterisessor* and *Gobius,* which are closely related, and *Pomatoschistus* belong to two distinct lineages (Agorreta et al., 2013), our results show support of a preserved effect of SDG content on sperm velocity in Gobiidae (Fig. S1). The SDG adaptations in Gobiidae possibly have an even older origin, as the sister families Butidae, Eleotridae and Odontobutidae also have SDG structures (Fishelson, 1991). Gobiidae is the second most species-rich vertebrate family known, and the most species-rich marine vertebrate family, with around 2000 described species, and 10 new species or more reported close to every year (Patzner et al., 2011). Their successful diversification and adaptation to spawning in fresh, brackish and marine water, in burrows and anadromously (Adrian-Kalchhauser et al., 2017), together with their potential as invasive species (Wonham et al., 2000) points to their ability to adapt into reproducing in novel environments. With SDGs being a conserved organ in gobies (Miller, 1984; Fishelson, 1991; Fig. S1), the ability to influence sperm function in the fertilization micro-environment is of interest for future research, in particular as this factor may contribute to their ability to spread into a range of environments.

Another example of a fish that modulates the direct environment of its spermatozoa is the three-spined stickleback (*Gasterosteus aculeatus*, Linnaeus 1758), which has ovarian fluid that enables its sperm to function in a range of salinities (Elofsson et al., 2003, 2006). Presumably, this function of the ovarian fluid has helped this species of stickleback to repeatedly colonize freshwater (Elofsson et al., 2003). Ovarian fluid has been shown to affect sperm function in several fish families (summarized in Elofsson et al., 2006), but to our knowledge, this is still uninvestigated in gobies. In gobies, eggs are attached to the substrate one-by-one (by an attachment network formed by a layer of filaments; Miller, 1984: Kramer and Patzner 2008), and the eggs appear ‘clean’. At this point, it is unknown if ovarian fluid might influence fertilization in gobies, alone or in combination with the SDG content studied here.

Gobies and their reproduction are studied as model organisms of sexual selection and evolutionary ecology (Locatello et al., 2007, 2013; Patzner et al., 2011; Svensson et al., 2017), and our results contribute to this growing body of literature. In conclusion, our study demonstrated that SDG content positively influences sperm velocity in the sand goby without affecting sperm viability*.* Whether or not the adaptation to alter the micro-environment of the sperm is widespread in the Gobiidae family, and how the trait is linked to their reproductive success, is still in need of investigation.

## MATERIALS AND METHODS

### Study species

Our experiments where conducted within the permit nr 86-2013 issued by the Ethical Committee for Animal Research in Gothenburg. Sand goby (*P. minutus*, Pallas 1770) males in were caught in Bökevik, Fiskebäckskil, Sweden (58°14'54.1″N 11°26'48.0″E), ∼10–11 May 2015. Males develop breeding colouration at the start of the breeding season (late April–early May), and in this study, we only used males in breeding colouration, as a way of avoiding immature males, as well as sneaker males, which lack breeding colouration (Kvarnemo et al., 2010). The males were kept in aquaria with a constant flow of natural seawater (salinity 32 PSU, temperature 10°C) and a 3 cm layer of fine sand for a maximum of 5 days. During this time, they were fed finely chopped food (a mix of frozen brown shrimp, mysid shrimp and Alaska pollock) *ad libitum*, once a day.

### Assay preparation

While a natural ejaculate may contain sperm and seminal fluid produced by the testes, potentially mixed with products of the SDG, we were interested in the specific impact of SDG content on sperm performance. For this reason, and because it is not possible to strip sand gobies without injuring the fish, we used dissections to be able to test this in a controlled way. Sample sizes were kept as low as possible and decided based on previous studies of sperm motility in the species (Svensson et al., 2017). Sperm from 10 males were tested using the following procedure: a male was given a blow to its head and then euthanized by destruction of the brain using a scalpel. Males were dissected and testes and sperm-duct glands were removed and separated within 1 min, using a dissection microscope (6× magnification, M3 Wild Heerbgrugg, Gais, Switzerland), stainless steel forceps and scissors (curved, sharp point, 4 inch, Sigma-Aldrich). The testes were placed into two separate 1.5 ml microcentrifuge tubes (Eppendorf, Hamburg, Germany), one testis without and one together with its SDG, creating the two different treatments: sperm only and sperm with SDG content. The organs were incised five times each using scissors (same model as above) and the content diluted with 60 μl calcium free Ringer's solution at 10°C (Karila et al., 1993) for a roughly double increase in volume. This Ringer's solution was chosen to prevent activation at this stage, and lack of activation was confirmed by visual inspection under a microscope with 10× magnification (AxioVert.A1, Carl Zeiss AG, Oberkochen, Germany). Release of molecules from the damaged tissue itself cannot be ruled out using this method, but was deemed insignificant due to the large differences in volume from between such a release and the emptying of the content of the glands and testes. Dilution of the sperm by the SDG content treatment was not found to be significant as sperm numbers did not differ between the treatments (Tables S1 and S2). The sample was then stirred using a Vortex (Vortex-Genie 2, Scientific Industries, Bohemia, NY, USA) three times for 1 s in rapid succession. The sperm were then activated by transferring 25 μl of each suspension to a new microcentrifuge tube, filled with 750 μl filtered seawater (salinity 32 PSU, temperature 10°C) using a micropipette (Transferpette, Brandtech, Essex, CT, USA) in an effort to mimic the natural way the ejaculate would mix with in the external environment. Thus, we had one tube for each treatment and male that was kept at 10°C in a thermostatic bath during the entire experiment except during vortexing. Testing of the samples was performed identically for both treatments.

### Definitions of measurements

We measured three traits that relate to sperm performance: velocity, motility and viability. Here we define them as follows: (1) velocity (or swimming speed) was only measured for sperm swimming at a minimum speed of 25 μm^−s^ (see Table S3 for settings). We measured velocity of the curvilinear path (VCL), velocity of the average path (VAP), velocity of the straight line path (VSL), progression (PROG) and beat cross frequency (BCF). We focused our analysis on VCL, due to its common use in studies on goby sperm velocity (Locatello et al., 2007), including the sand goby (Svensson et al., 2017) and because it has been found to correlate with fertilization success across taxa (Casselman et al., 2006; Fitzpatrick et al., 2012) including gobies (Locatello et al., 2013). Results for VAP, VSL, PROG and BCF, as well as number of sperm tracked (N) are reported in Table S1. (2) Motility was measured as percent sperm that exceeded minimum speed (same as above). (3) Viability was calculated as percentage live sperm (number of live/number of live and dead ×100), measured after 10 min and 24 h.

### Sperm velocity and motility measurements

To assess sperm velocity and percentage of motile sperm, the sample for each male and treatment was stirred using a Vortex-Genie 2 (same model as above) 3×1 s to mix it. From this, three 45 μl suspensions were transferred to an albumin coated microscope slide fitted with three O-rings. Each drop was covered with an albumin coated coverslip to form a suspended drop following established protocols (Havenhand and Schlegel, 2009; Havenhand et al., 2008; Schlegel et al., 2012). This procedure was replicated twice for a total of six technical replicates per male and treatment. For each male, we randomized if we analysed the tube containing sperm with or without SDG content first. Using a high-speed camera (PixeLINK PL-D725, Ottawa, Ontario, Canada) fitted to a differential interference contrast microscope (AxioVert.A1, Carl Zeiss AG, Oberkochen Germany), each drop was filmed at 10× magnification, standard contrast and illumination, for 15 frames (30 frames s^−1^, size 2592*2048 pixels, exposure time 10 ms, Gain 0, Gamma 0.1). All handling was done as quickly as possible and always <10 min after dissection.

### Sperm viability measurements

To investigate sperm viability the following procedure was done 10 min (±1 min) after organ dissection and 24±1 h later for each treatment: 100 μl was taken from the sample for each male and treatment and transferred to a separate 600 μl microcentrifuge tube (Eppendorf, Hamburg, Germany) and vortexed for 3×1 s. This sample was stained with 1 μl diluted SYBR14 (1 parts SYBR14 to 49 parts DMSO) and then stained with propidium iodide (diluted one parts propidium iodide to four parts with DMSO) (LIVE/DEAD^®^ Sperm Viability Kit, L7011, Life Technologies, Thermo Fisher Scientific) (Garner and Johnson, 1995). The sample was then vortexed again for 3×1 s before 25 μl was transferred to a microscope slide and allowed to spread into a thin film to minimize the depth of field and avoid excess movement. The microscope was then focused on the glass surface and an image taken first illuminated with green light (Cy 3 filter, 520-560 nm), then blue light (GFP filter, 450-490 nm), using the following camera settings: size 2592×2048 pixels, exposure time 500 ms, gain 18.06, gamma 4. This procedure was replicated for the three stained samples on the same slide, resulting in three technical replicates per male and treatment. For each male, we randomized if we analysed the tube containing sperm with or without SDG content first.

### Data analysis

Images were digitally filtered and analysed using ImageJ (National Institutes of Health) and videos were analysed using a computer assisted sperm analysis plugin (CASA) (Wilson-Leedy and Ingermann, 2007, 2011). See Table S3 for details on settings used. An average of 282 sperm cells per film were tracked.

Data were statistically analysed using SPSS 22 (IBM) and R (3.5.1, R Foundation). All data were tested for outliers, normal distribution and homogeneity of variances and covariance. There were no outliers in the data (as assessed by box-plot) and data was normally distributed (as assessed by Q-Q plot and Shapiro-Wilk test for normality) but assumptions of homogeneity of variance were not met for viability (Levene's Test, *F*_1,19_=7.23, *P*=0.015) or VCL (Levene's Test, *F*_1,108_=5.039, *P*=0.027). Sperm motility data were analysed by linear mixed effects models, using ‘treatment’ (sperm with or without SDG content) as fixed factor and controlling for individual variation by including ‘individual’ and ‘technical replicate’ as random factors. To obtain the effect size estimate (*P*-value), a likelihood ratio test was then used to compare two mixed effects models, one with and the other without the ‘treatment’ factor.

## Supplementary Material

Supplementary information
