# Supplementary material for: Sperm-duct gland content increases sperm velocity in the sand goby
Source: Biol Open. 2019 Mar 5;8(3):bio037994. doi: 10.1242/bio.037994 (PMC6451343; doi:10.1242/bio.037994)
Supplement: Supplementary information [file biolopen-8-037994-s1.pdf]

Table S1. Statistical results from Linear Mixed Models on the CASA parameters generated in the study. Statistics presented from linear mixed models in constructed using the package lme4 in R (3.5.1, R Foundation).

**Threshold for detection in CASA**	*X^2*	*P*	
Model statistics	0.8671	0.3518	
Random effects:	*Variance*	*Std.Dev.*	
Individual	0.003856	0.0621	
Replicate	0.000000	0.0000	
Residual	0.029404	0.1715	
Fixed effects:	*Estimate*	*Std. Error*	*t-value*
(Intercept)	0.58761	0.03072	19.126
Treatment	-0.03062	0.03278	-0.934

**Percent motile sperm**	*X^2*	*P*	
Model statistics	0.1374	0.7109	
Random effects:	*Variance*	*Std.Dev.*	
Individual	0.0132172	0.11497	
Replicate	0.0002177	0.01476	
Residual	0.0102185	0.10109	
Fixed effects:	*Estimate*	*Std. Error*	*t-value*
(Intercept)	0.233728	0.039411	5.931
Treatment	0.007183	0.019348	0.371

**VCL (Velocity of the curvilinear path)**	*X^2*	*P*	
Model statistics	5.475	0.01929	
Random effects:	*Variance*	*Std.Dev.*	
Individual	91.74	9.578	
Replicate	1.73	1.315	
Residual	101.02	10.051	
Fixed effects:	*Estimate*	*Std. Error*	*t-value*
(Intercept)	75.125	3.375	22.259
Treatment	-4.564	1.924	-2.373

**VAP (Velocity of average path)**	*X^2*	*p*	
Model statistics	7.7964	0.005235	
Random effects:	*Variance*	*Std.Dev.*	
Individual	120.9	11.00	
Replicate	0.0	0.00	
Residual	144.2	12.01	
Fixed effects:	*Estimate*	*Std. Error*	*t-value*
(Intercept)	60.502	3.852	15.707
Treatment	-6.537	2.297	-2.846

Biology Open • Supplementary information

**VSL (Velocity of straight line path)**	*X^2*	*P*	
Model statistics	7.7992	0.005227	
Random effects:	*Variance*	*Std.Dev.*	
Individual	8.009e+01	8.949e+00	
Replicate	9.596e-14	3.098e-07	
Residual	1.014e+02	1.007e+01	
Fixed effects:	*Estimate*	*Std. Error*	*t-value*
(Intercept)	48.955	3.153	15.527
Treatment	-5.484	1.927	-2.846

**PROG (Progression)**	*X^2*	*P*	
Model statistics	7.8999	0.004944	
Random effects:	*Variance*	*Std.Dev.*	
Individual	741.6	27.23	
Replicate	0.0	0.00	
Residual	911.3	30.19	
Fixed effects:	*Estimate*	*Std. Error*	*t-value*
(Intercept)	148.691	9.566	15.543
Treatment	-16.549	5.776	-2.865

**BCF (Beat cross frequency)**	*X^2*	*P*	
Model statistics	2.0632	0.1509	
Random effects:	*Variance*	*Std.Dev.*	
Individual	5.283e-01	7.268e-01	
Replicate	7.595e-20	2.756e-10	
Residual	9.785e-01	9.892e-01	
Fixed effects:	*Estimate*	*Std. Error*	*t-value*
(Intercept)	6.7842	0.2673	25.378
Treatment	0.2732	0.1892	1.443

Biology Open • Supplementary information

Table S2. CASA results from Sperm Only and Sperm with SDG content treatments. Treshold = threshold for detection in ImageJ, % mot = percentage motile sperm in sample, VCL = velocity of the curvilinear path, VAP = velocity of the average path, VSL = velocity of the straight line, PROG = progression (average distance in µm of the sperm from its origin on the average path during all frames analysed), BCF = beat cross frequency. See reference (Wilson-Leedy & Ingermann, 2011) in manuscript for detailed descriptions of measurements.

Sperm Only	Threshold	Sperm motility (%)	VCL (µm -s)	VAP (µm -s)	VSL (µm -s)	PROG (µm -s)	BCF	Number of tracked sperm
Mean	0.560	0.243	70.582	53.845	43.366	131.817	7.069	315.727
s.d.	0.124	0.117	9.051	10.620	8.710	26.690	0.904	220.516
s.e.m.	0.039	0.037	2.862	3.358	2.754	8.440	0.286	69.733
**Sperm w. SDG content**								
Mean	0.585	0.233	74.887	60.151	48.650	147.805	6.788	257.187
s.d.	0.128	0.140	11.696	15.733	13.060	39.264	0.904	123.885
s.e.m.	0.041	0.044	3.699	4.975	4.130	12.416	0.286	39.176

Table S3. Settings used during tracking analysis for Sperm Tracker in CASA for ImageJ. Table shows order of settings as alphabetical characters, specific setting, units in brackets and responding value used during analysis. Software available from https://imagej.nih.gov/ij/plugins/casa.html during time of publishing.

Sperm Tracker Settings	*Value*
a, Minimum sperm size (pixels):	2
b, Maximum sperm size (pixels):	40.0
c, Minimum track length (frames):	15
d, Maximum sperm velocity between frames (pixels):	20
e, Minimum VSL for motile (um/s):	15
f, Minimum VAP for motile (um/s):	20.0
g, Minimum VCL for motile (um/s):	25.0
h, Low VAP speed (um/s):	25
i, Maximum percentage of path with zero VAP:	1.0
j, Maximum percentage of path with low VAP:	50
k, Low VAP speed 2 (um/s):	20.0
l, Low VCL speed (um/s):	25.0
m, High WOB (percent VAP/VCL):	80.0
n, High LIN (percent VSL/VAP):	80.0
o, High WOB two (percent VAP/VCL):	80.0
p, High LIN two (percent VSL/VAP):	80.0
q, Frame Rate (frames per second):	30.0
r, Microns per 1000 pixels:	480.0

Biology Open • Supplementary information

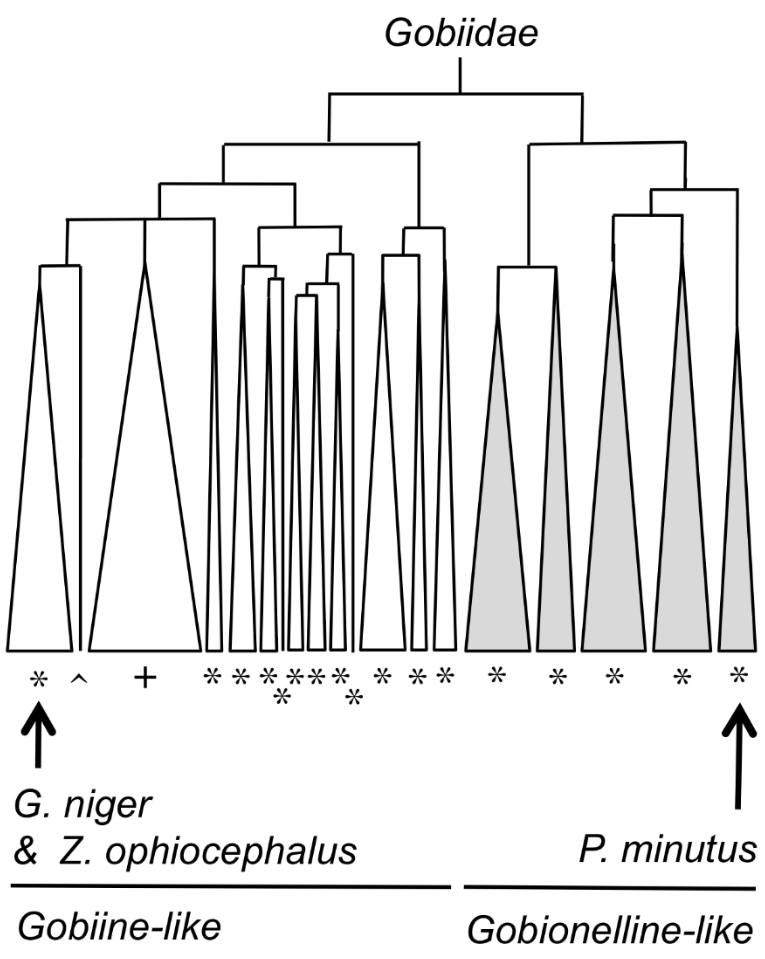

Figure S1. SDGs in the Gobiidae family tree.

Phylogeny of Gobiidae (redrawn from Agorreta et al., 2013) with placement of *Pomatoschistus minutus* (this study), *Gobius niger* and *Zosterisessor ophiocephalus* (also found to exhibit velocity-boosting SDG content (Locatello et al., 2013; Poli et al., 2018)). The species occur in the Gobionelline-like (in gray to highlight the study species placement and aid with visual separation) and Gobiine-like subfamilies, respectively. Asterisks show lineages where sperm duct glands have been described (Fishelson, 1991). The Kraemeria- (symbol ‹) and Gobiosomatini-lineages (symbol +) have not been morphologically studied with regards to sperm duct glands.

Biology Open • Supplementary information
